# Initial solution pH value for the construction of a 3D hydroxyapatite via the trisodium citrate-assisted hydrothermal route

**DOI:** 10.3389/fchem.2024.1442824

**Published:** 2024-07-18

**Authors:** Mei-li Qi, Wen Wang, Xiao-Cun Liu, Xiaoying Wang, Jin Li, Haijun Zhang

**Affiliations:** ^1^ School of Transportation Civil Engineering, Shandong Jiaotong University, Ji’nan, China; ^2^ Shanghai Tenth People’s Hospital, School of Medicine, Tongji University, Shanghai, China

**Keywords:** three-dimensional hydroxyapatite, hydrothermal synthesis, initial pH, trisodium citrate, morphology

## Abstract

In this study, a trisodium citrate (TSC)-assisted hydrothermal method is utilized to prepare three-dimensional hydroxyapatite (3D HA). Understanding the role of TSC in the preparation of 3D HA crystals may provide valuable methods to design advanced biomaterials. As one of the indexes of solution supersaturation, the initial pH (ipH) value can not only directly affect the nucleation rate, but also affect the growth of HA crystals. In this work, the effect of the ipH on the microstructure, particle size distribution, and specific surface area of the 3D HA is explored. Results showed that the morphology of 3D HA transformed from a bundle to a dumbbell ball and then a dumbbell with an increase in the ipH. A corresponding mechanism of such a structural evolution was proposed, providing inspiration for the fabrication of innovative 3D HA structures with enhanced biological functionality and performance.

## 1 Introduction

Naturally occurring biominerals and biomolecules within biological organisms typically possess innovative three-dimensional (3D) structures and distinctive biological functions, such as the specific 3D structure of dental enamel (apatite) (Wang et al., 2011; Grandfield et al., 2022; Yu and Zhu, 2024). As an important calcium phosphate biomineral frequently found in mammalian hard tissues, hydroxyapatite (HA) has become a focal point of research due to its excellent biocompatibility, favorable bioactivity, and chemical composition similar to human bone tissue (Zhou and Lee, 2011; Shi et al., 2018; Xu et al., 2023). HA with 3D structures have wide potential applications in the field of drug delivery systems and ion/heavy metal absorption agents due to the rough surface and highly specialized surface area (Yang et al., 2013a; Zeng et al., 2019; Liu et al., 2020; Wang et al., 2024). Therefore, the facile synthesis of 3D HA is crucial, and great efforts have been made to prepare such structures (Mocioiu et al., 2019; Xu et al., 2022).

Among the many routes for synthesizing 3D HA, the hydrothermal method is favored for its ability to produce high-purity, well-crystallized HA at relatively low temperatures and pressures (Hao et al., 2014; Lei et al., 2020; Jiang et al., 2021). In recent years, researchers have been able to further control the microstructure and morphology of HA by introducing trisodium citrate (TSC) as an auxiliary agent during the hydrothermal process (Yang et al., 2012; Qi et al., 2023). The employment of a TSC-assisted hydrothermal method for the synthesis of 3D HA has opened new avenues for the development of advanced biomaterials. Recognizing the pivotal role of TSC in the crystallization process of HA is essential, as it can significantly influence the resulting material’s properties and potential applications in the biomedical field.

The initial pH (ipH) value, a key indicator of solution supersaturation, plays a dual role in the synthesis process by influencing both the nucleation rate and the subsequent growth behavior of HA crystals (Wang et al., 2021). Adjustments to the ipH can alter the ion concentration and reaction kinetics in the solution, thereby affecting the size, shape, and crystallinity of HA crystals (Kalpana and Nagalakshmi, 2023; áM Gan et al., 1999). Overly low ipH values may lead to impurities, while overly high ipH values may cause the formation of lath-like HA crystals (Zhou and Lee, 2011; Sadat-Shojai et al., 2012). Therefore, an in-depth study of the impact of the ipH on the synthesis of 3D HA via TSC-assisted hydrothermal methods is of great significance for optimizing the synthesis process and achieving ideal material properties. Although the synthesis of HA has been extensively studied, discovering a novel approach to synthesize 3D HA at low ipH values presents a significant challenge (Dorozhkin, 2011; Šupová, 2015; Szcześ et al., 2017; Haider et al., 2017; Mohd et al., 2020).

In the current work, 3D HA through TSC-assisted hydrothermal methods under low ipH conditions is synthesized. The influence of the ipH on various aspects of the synthesized HA, including its morphology, structure, particle size distribution, and specific surface area, is thoroughly investigated. By comparing HA samples synthesized under different ipH values, the control mechanism of the ipH on the morphology of HA synthesis is revealed, providing a theoretical foundation and experimental guidance for the design and preparation of HA-based biomaterials with specific application functions.

## 2 Materials and methods

### 2.1 Materials

The anhydrous calcium chloride (CaCl_2_, AR), diammonium hydrogen phosphate ((NH_4_)_2_HPO_4_, AR), TSC (AR), nitric acid (HNO_3_, AR) and urea (CO(NH_2_)_2_, AR) for experimental use were purchased from the Sinopharm Chemical Reagent Company Limited of China (Shanghai, China). All of the chemical reagents were used directly without further purification.

### 2.2 Preparation

A typical product was synthesized as follows: 0.06 mol/L (NH_4_)_2_HPO_4_, 0.01 mol/L CaCl_2_ and 1 mol/L urea aqueous solutions were mixed thoroughly. The ipH of the mixture was adjusted to 2.5, 3.0, and 3.5 using a dilute HNO_3_ solution under magnetic stirring. After this, the TSC was added, and the TSC/Ca molar ratio was maintained at 1.0. The mixture was then hydrothermally treated in a Teflon-lined autoclave at 180°C for 10 h. Finally, the obtained resultants were washed with deionized water and ethanol, centrifuged, and dried.

### 2.3 Characterization

An X-ray diffraction (XRD, Bruker D8 Advance, CuKα radiation, λ = 1.5418 Å) and Fourier transform infrared spectroscopy (FTIR, Nicolet IS50) were utilized to identify the phase composition and functional groups of the products. A field scanning electron microscope (FE-SEM, JSM-7610F, 5 kV) and a transmission electron microscope (TEM, JEM2100Plus, 200 kV) were used to observe the morphology and microstructure of the samples. The powder was sputter-coated with gold before the FE-SEM tests due to their nonconductivity. A laser particle size analyzer (Mastersizer 2000) was used to evaluate the particle size distribution (PSD). Prior to the TEM and PSD tests, the powder was dispersed ultrasonically in anhydrous ethanol for 10 min. The Brunauer-Emmett-Teller (BET) surface area were measured using a Micromeritics ASAP 2460 instrument.

## 3 Results and discussion

### 3.1 Phase and functional analysis

**FIGURE 1:**
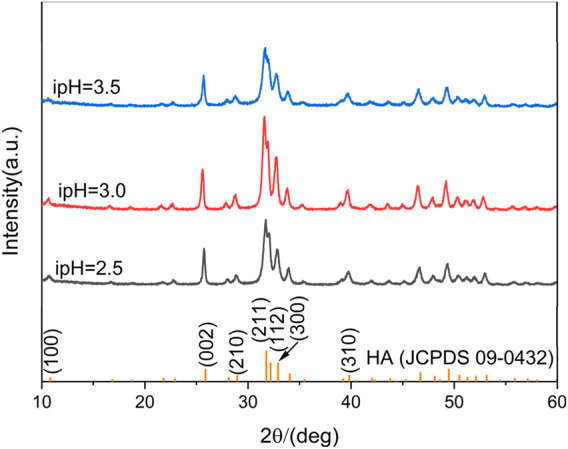
XRD patterns of the products prepared under the different ipHs.

The phase composition of the synthesized products under various ipHs were characterized using XRD tests, and the results are shown in Figure 1. A comparison with the standard HA profile (JCPDS 09-0432), depicted as the purple bar chart in Figure 1, confirmed that all of the synthesized products corresponded to the pure HA phase. The difference was in the intensity of the diffraction peaks, reflecting the different crystallinity of the HA products (Guerra-López et al., 2024). With an increase in the ipH from 2.5 to 3.0, the supersaturation of Ca^2+^ and PO_4_^3-^ in the reaction system increases, and it is easier to generate the crystalline phase HA (Wang et al., 2021). Therefore, the intensity of the crystal plane diffraction peak tended to increase. However, when the solution reached saturation, the crystallinity degree of the HA sample decreased (ipH = 3.5).

**FIGURE 2:**
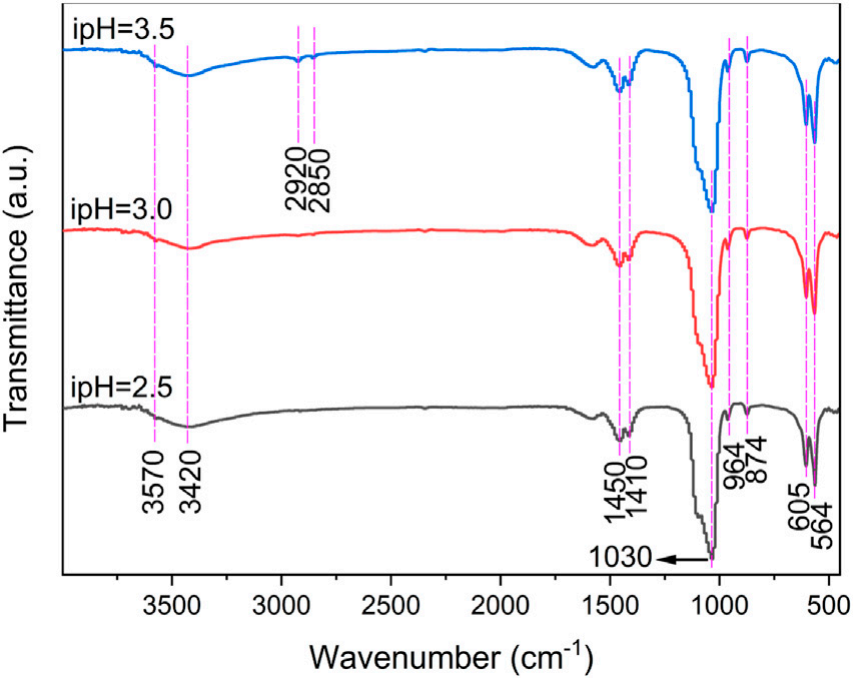
FTIR spectra of the products prepared under the different ipHs.

The FTIR tests of the synthesized products under various ipHs were consistent with the XRD patterns, since all the spectra were similar (Figure 2). Each absorption peak in the FTIR spectrum reflected the vibration mode of each molecular group in the HA crystals. The absorption peaks at 3420 and 3570 cm^-1^ corresponded to water molecules adsorbed on the sample surface and the stretching vibration absorption mode of the OH^−^ groups, respectively (Sun et al., 2016). The peaks at 1030 and 964 cm^-1^ and 605 and 564 cm^-1^ were attributed to the asymmetric stretching vibration and bending vibration of the PO_4_^3-^ group (Yin-Chuan et al., 2022; Ferrairo et al., 2023), respectively. The peaks observed in the range at 2920 and 2850 cm^-1^ are assigned to the C-H vibrations (Dutta, 2017). The double bands at 1450 and 1410, as well as 874 cm^-1^, indicated the B-type substitution of the PO_4_^3-^ group in HA by CO_3_^2-^ that was produced by the hydrolysis of urea above 80°C (Xu et al., 2022). Such results further indicated that the HA products were carbonated HA, similar to the primary inorganic component of bone mineral (Kumar et al., 2019).

### 3.2 Microstructural characterization

**FIGURE 3:**
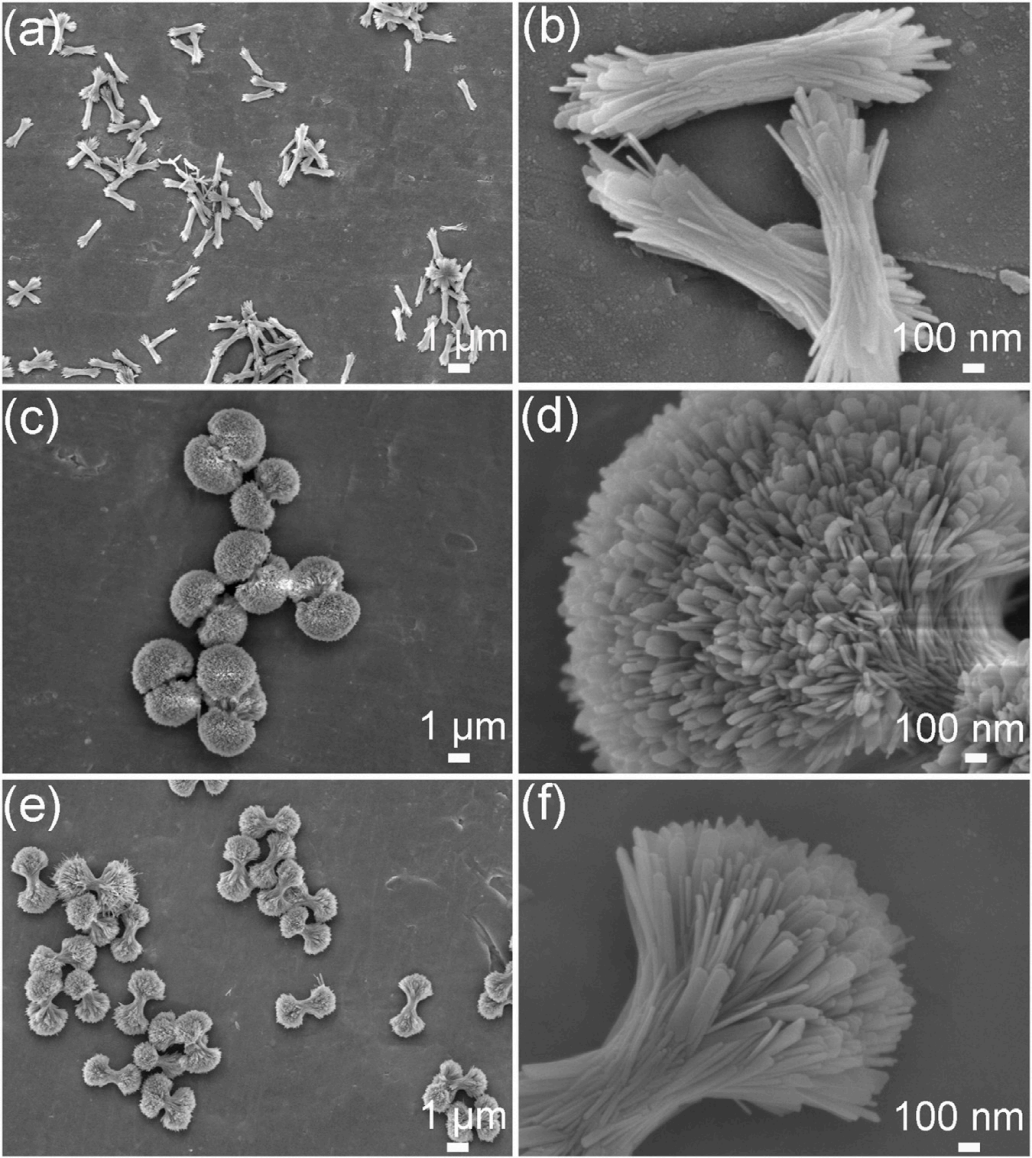
FE-SEM images of the HA products prepared under the different ipHs. **(A,B)** ipH = 2.5, **(C,D)** ipH = 3.0, and **(E,F)** ipH = 3.5.

The morphological evolutions of the HA products prepared under the different ipHs as characterized by FE-SEM are displayed in Figure 3. Initially, bundle-like HA crystals with lengths of approximately 1 μm (Figure 3A) were obtained when the ipH was 2.5. As the ipH increased to 3.0, the HA products consisted of dumbbell balls (Figure 3C). However, with a further increase in the ipH to 3.5, the morphology of the HA transformed into dumbbells (Figure 3E). High magnification observations suggest that the constituent units of the HA crystals exhibit a sheet-like structure (Figures 3B, D, F). This morphological progression was indicative of the possible intricate interplay between the ipH and the crystallization kinetics during the hydrothermal synthesis process (Zhang and Darvell, 2011).

**FIGURE 4:**
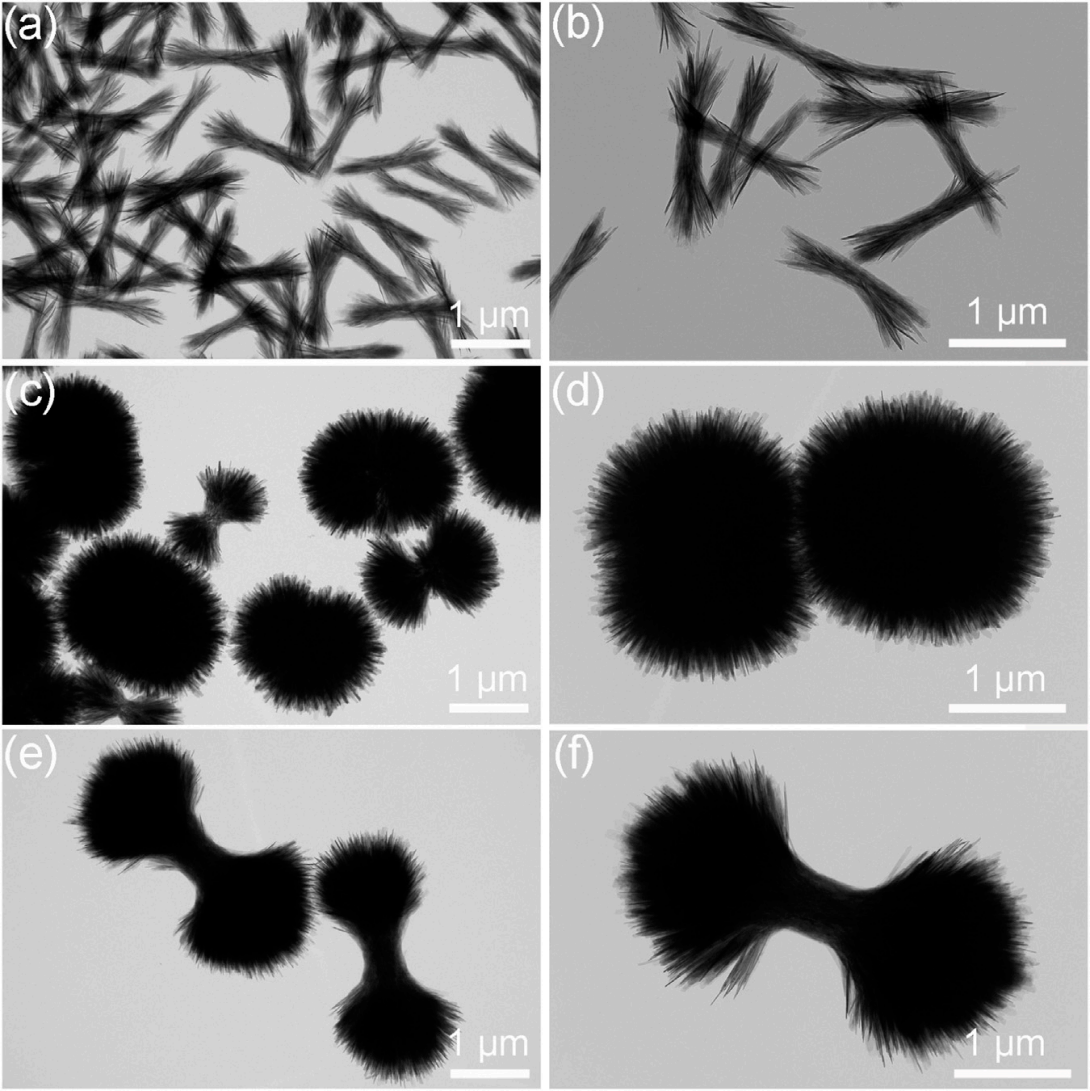
TEM images of the HA products prepared under the different ipHs. **(A,B)** ipH = 2.5, **(C,D)** ipH = 3.0, and **(E,F)** ipH = 3.5.

The microstructural evolutions of the HA products prepared under the different ipHs as characterized by TEM are displayed in Figure 4. The morphology undergoes a process from bundle-like (Figures 4A, B), to dumbbell balls (Figures 4C, D) and then dumbbells (Figures 4E, F). Figure 4 illustrates the microstructural evolution of HA products prepared under varying initial pH conditions, as characterized by TEM. The morphology transitions from a bundle-like form (Figures 4A, B), through dumbbell-shaped structures (Figures 4C, D), and finally to dumbbell-like configurations (Figures 4E, F).

The final morphology of the synthesized HA depended on the relative growth rate of each crystal surface of the HA. HA is a weak alkaline calcium phosphate salt, and the entire reaction and ion adsorption were slower at a lower pH value. Under the control of the TSC, the HA grew in a divergent manner, and the dumbbell ball-shaped HA formed.

### 3.3 Particle size distribution and specific surface area

**FIGURE 5:**
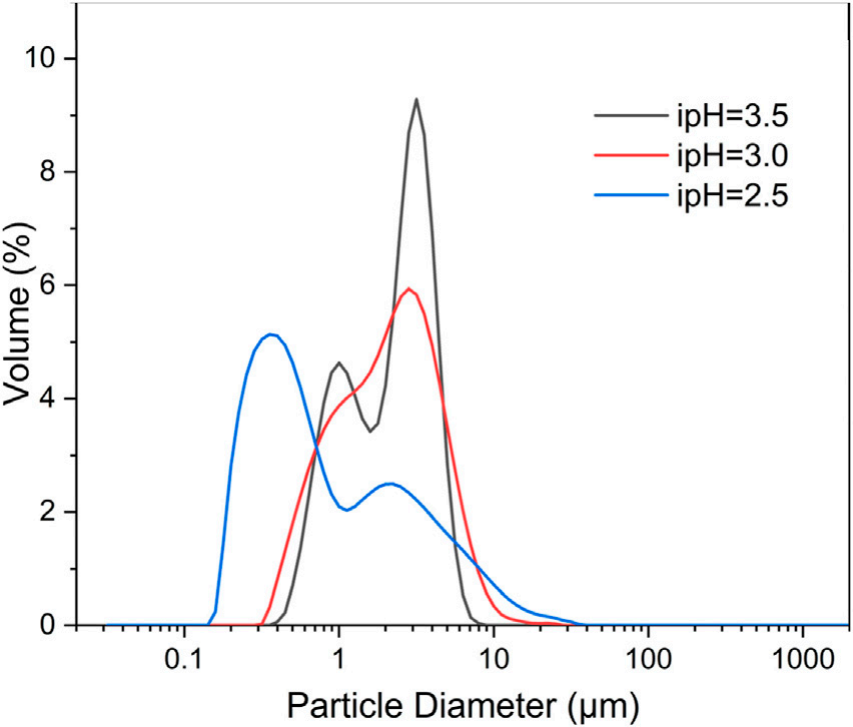
Particle size distributions of the three-dimensional HA prepared under the different ipHs.

The particle size distribution (PSD) and specific surface area are crucial parameters that significantly affect the characteristics of 3D HA (Qi et al., 2016). As depicted in the PSD curve (Figure 5), it is apparent that the peak of the HA sample synthesized at an ipH of 2.5 exhibited a shift to the left compared to the other two. Additionally, the sample prepared at an ipH of 3.0 aligned optimally with a normal distribution. The majority of the 3D HA crystals fell within a size range of 0.1–10 µm.

Furthermore, particle sizes and BET specific surface area of the synthesized three-dimensional HA under different ipHs are counted (Table 1). The mean particle size of the HA products underwent a dual trend of increase and subsequent decrease as the ipH values rose, and the HA dumbbell-shaped particles prepared at an ipH of 3.0 had the largest dimensions (2.45 µm). The BET specific surface area of the HA products mirrored this biphasic trend, increasing and then decreasing as the ipH values increased, as depicted in Table 1. A comparison of all the samples showed that the HA dumbbell-shaped particles prepared at an ipH of 3.0 had the highest specific surface areas, at 45.35 m^2^/g. These exceptional properties render it an ideal candidate for applications as drug-delivery carriers, offering promising potential in the field of controlled drug release and targeted therapy.

**TABLE 1:** Particle sizes and specific surface areas of the synthesized three-dimensional HA under different ipHs.

ipHs	d0.1 (μm)	d0.5 (μm)	d0.9 (μm)	Mean size (μm)	BET surface area (m^2^/g)
2.5	0.24	0.63	4.67	1.82	41.93
3.0	0.65	1.99	4.78	2.45	45.35
3.5	0.76	2.26	3.97	2.29	42.95

Note: d0.1, d0.5 and d0.9 means 10%, 50% and 90% of total particle size less than some value, respectively.

### 3.4 Formation mechanism of 3D HA under the different hydrothermal ipHs by TSC

**FIGURE 6:**
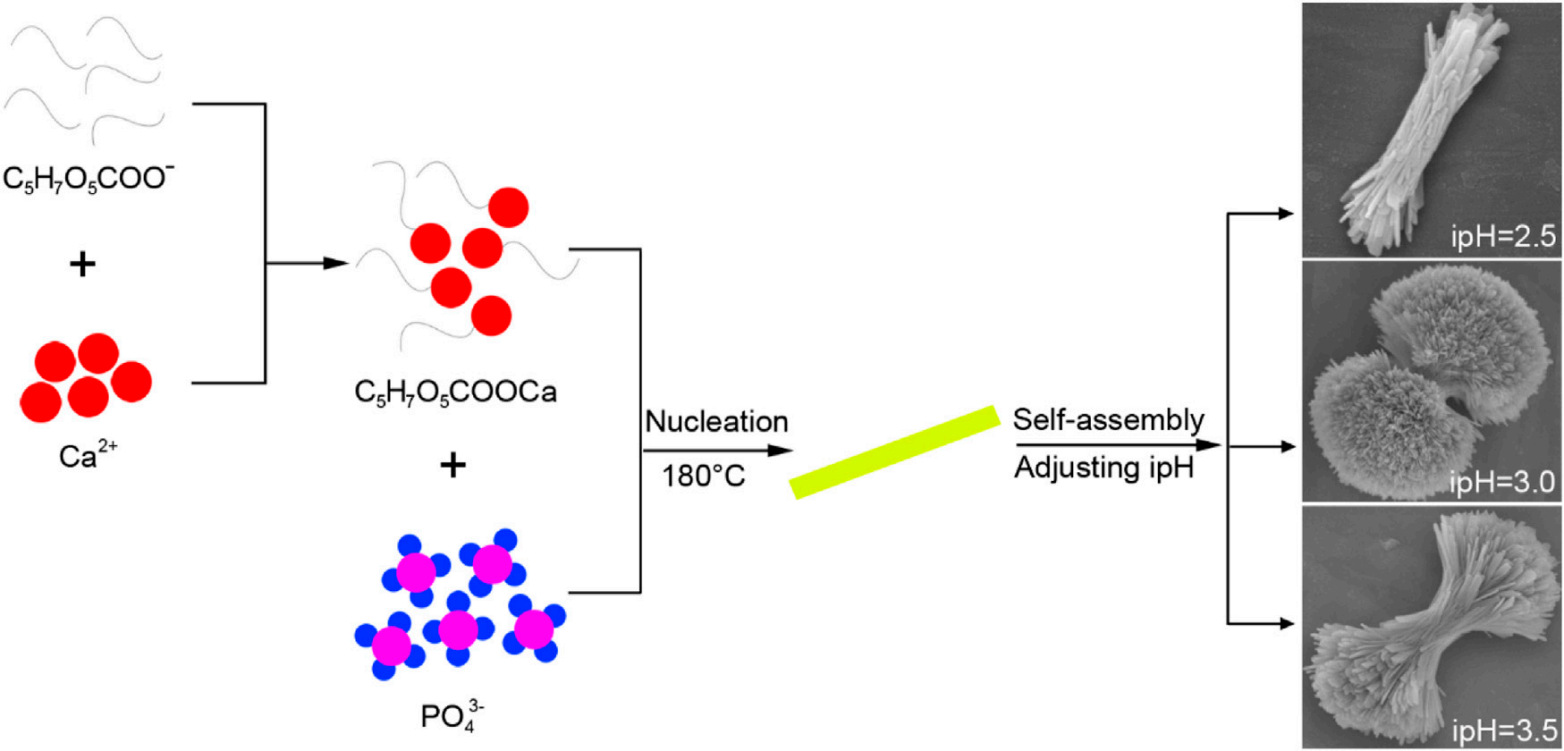
Formation mechanism of the three-dimensional HA prepared under the different ipHs via a the TSC-assisted hydrothermal route.

IpH serves as a crucial indicator of supersaturation and indirectly influences the rate of crystal nuclei formation that in turn affects the growth of HA crystals (Akhtar and Pervez, 2021). To demonstrate the 3D HA prepared under the different ipHs via a TSC-assisted hydrothermal route, a possible mechanism was proposed (Figure 6). During the initial stages of the synthesis process, citrate interacted with Ca^2+^ ions in the reactive system to form soluble calcium citrate complexes This chelation reaction can occur through the three carboxylate groups (pKa_1_ = 3.14, pKa_2_ = 4.77, pKa_3_ = 6.39) for citrate and inhibites the direct precipitation of HA (Chu et al., 2011). Moreover, the presence of citrate can modulate the surface charge of the emerging crystals, thereby influencing the anisotropic growth patterns on the HA crystal surfaces, as indicated by previous research (Yang et al., 2013b). As the ipH gradually increased, the decomposition rate of the Ca-TSC complex accelerated, leading to the release of Ca^2+^ ions that could participate in the crystallization process. Concurrently, the hydrolysis of urea generated hydroxide ions (OH^−^) and, subsequently, carbonate ions (CO_3_^2-^) when the hydrothermal temperature reached 80°C. Under these conditions, plate-like carbonated HA crystal nuclei begin to form, and as the ipH was 2.5 which is right below the pKa_1_ for citrate, they aggregated to evolve into bundle-like crystallites. The drive to minimize the overall surface energy prompted the bundle-like crystallites to grow in size and self-assemble, eventually forming dumbbell-shaped clusters when the ipH rose to 3.0, nearly at the pKa_1_ for citrate. This morphological transformation was attributed to the increased crystallinity and the specific arrangement of the crystallites to reduce the surface energy and achieve a more stable configuration, just as the formation of dumbbell fluorapatite aggregates earlier reported by Busch et al. (Busch et al., 1999). However, when the ipH reached 3.5 which is just above the pKa_1_ for citrate, the solution became highly supersaturated, leading to an abundance of bundle-like HA crystallites that did not readily coalesce into dumbbell clusters. As the ipH increases, the final pH values after the reaction are 9.08, 8.91, and 8.89, respectively, indicating a negligible change. These values align with the calcium-to-phosphorus (Ca/P) molar ratios detailed in the Preparation section. Based on the comprehensive analysis presented, maintaining the ipH at 3.0 was deemed favorable for the formation of the HA dumbbell structures.

This proposed mechanism offers valuable insights into the intricate interplay between ipH, TSC, and the crystallization kinetics of HA. By understanding and controlling these factors, it becomes possible to tailor the synthesis of 3D HA, which is essential for the development of advanced biomaterials with specific properties.

## 4 Conclusion

In summary, three-dimensional HA with an average diameter of 2.45 μm and a BET surface area of 42.95 m^2^/g were successfully prepared via a TSC-assisted hydrothermal route. The influence of the ipH on the morphologies of the hydrothermally synthesized HA crystals was also investigated. As the ipH value increased, the resulting HA morphologies underwent a systematic evolution, transitioning from bundled structures to a dumbbell-shaped forms and eventually settling into dumbbell morphologies. The optimum ipH for the development of 3D HA with a desirable mean particle size and BET specific surface area was 3.0. To elucidate the formation of 3D HA particles under varying ipH conditions through the TSC-assisted hydrothermal route, a plausible mechanism was proposed. This work not only sheds light on the underlying processes that governs the synthesis of 3D HA, but also provides valuable insights for the design and optimization of advanced HA materials with tailored properties for biomedical applications.

## Data Availability

The original contributions presented in the study are included in the article/Supplementary Material, further inquiries can be directed to the corresponding authors.
